# Is waist-to-height ratio a useful indicator of cardio-metabolic risk in 6-10-year-old children?

**DOI:** 10.1186/1471-2431-13-91

**Published:** 2013-06-11

**Authors:** Valesca Mansur Kuba, Claudio Leone, Durval Damiani

**Affiliations:** 1 Endocrinologist at the Reference Center for the Treatment of Children and Adolescents, Campos, Rio de Janeiro, Brazil; 2 Diabetes and Endocrinology Institute of Rio de Janeiro (IEDE), Rio de Janeiro, Brazil; 3 Department of Mother and Child Health, School of Public Health, University of São Paulo, São Paulo, Brazil; 4 Head of the Pediatric Endocrinology Unit, Instituto da Criança, University of São Paulo Medical School, São Paulo, Brazil; 5 Rua Siqueira Campos,112, Campos, 28010-015 Rio de Janeiro, Brazil

**Keywords:** Waist-to-height ratio, Obesity, Insulin resistance, Cardiovascular disease, Body mass index

## Abstract

**Background:**

Childhood obesity is a public health problem worldwide. Visceral obesity, particularly associated with cardio-metabolic risk, has been assessed by body mass index (BMI) and waist circumference, but both methods use sex-and age-specific percentile tables and are influenced by sexual maturity. Waist-to-height ratio (WHtR) is easier to obtain, does not involve tables and can be used to diagnose visceral obesity, even in normal-weight individuals. This study aims to compare the WHtR to the 2007 World Health Organization (WHO) reference for BMI in screening for the presence of cardio-metabolic and inflammatory risk factors in 6–10-year-old children.

**Methods:**

A cross-sectional study was undertaken with 175 subjects selected from the Reference Center for the Treatment of Children and Adolescents in Campos, Rio de Janeiro, Brazil. The subjects were classified according to the 2007 WHO standard as normal-weight (BMI *z* score > −1 and < 1) or overweight/obese (BMI *z* score ≥ 1). Systolic blood pressure (SBP), diastolic blood pressure (DBP), fasting glycemia, low-density lipoprotein (LDL), high-density lipoprotein (HDL), triglyceride (TG), Homeostatic Model Assessment – Insulin Resistance (HOMA-IR), leukocyte count and ultrasensitive C-reactive protein (CRP) were also analyzed.

**Results:**

There were significant correlations between WHtR and BMI *z* score (*r* = 0.88, *p* < 0.0001), SBP (*r* = 0.51, *p* < 0.0001), DBP (*r* = 0.49, *p* < 0.0001), LDL (*r* = 0.25, *p* < 0.0008, HDL (*r* = −0.28, *p* < 0.0002), TG (*r* = 0.26, *p* < 0.0006), HOMA-IR (*r* = 0.83, *p* < 0.0001) and CRP (*r* = 0.51, *p* < 0.0001). WHtR and BMI areas under the curve were similar for all the cardio-metabolic parameters. A WHtR cut-off value of > 0.47 was sensitive for screening insulin resistance and any one of the cardio-metabolic parameters.

**Conclusions:**

The WHtR was as sensitive as the 2007 WHO BMI in screening for metabolic risk factors in 6-10-year-old children. The public health message “keep your waist to less than half your height” can be effective in reducing cardio-metabolic risk because most of these risk factors are already present at a cut point of WHtR ≥ 0.5. However, as this is the first study to correlate the WHtR with inflammatory markers, we recommend further exploration of the use of WHtR in this age group and other population-based samples.

## Background

Obesity is subclinical inflammation characterized by the secretion of cytokines that influence the formation of atherosclerotic plaque and endothelial dysfunction [1]. This inflammatory process begins in childhood and involves leukocytes and the hepatic secretion of C-reactive protein (CRP), whose serum levels are significantly correlated with abdominal adiposity in adolescents [2]. Although the relationship between abdominal obesity and cardio-metabolic disorders has already been demonstrated through the assessment of the abdominal circumference in adults, in children there are controversies. For some authors [3] body mass index (BMI) and waist circumference (WC) are equal indicators of metabolic risk, whereas others believe WC to be a better indicator [4]. Because both WC and BMI depend on the use of sex- and age-specific percentile tables in children, waist-to-height ratio (WHtR) has emerged as new option that is more attractive than these other anthropometric indices. WHtR is easier to obtain, does not require reference tables, is less influenced by sexual maturity, is suitable for population and epidemiologic studies and can be used on a large scale in screening for metabolic risk in both adults and children [5,6].

The objective of this study is to compare the WHtR to the 2007 World Health Organization (WHO) reference for BMI in screening for the presence of cardio- metabolic and inflammatory risk factors in normal-weight and overweight/obese 6–10-year-old children.

## Methods

This was a cross-sectional study of a sequential sample of children from the Pediatric Endocrinology outpatient clinic of the Reference Center for the Treatment of Children and Adolescents (CRTCA) in Campos, State of Rio de Janeiro, in the southeast of Brazil. Data were collected between November 2006 and April 2008. The sample comprised 175 children of both sexes aged 6–10. All subjects were classified as Tanner staging as pre-adolescents, with no apparent secondary sexual characteristic. Most participants were from low-income families and many were of African American descent. Although puberty may start earlier in African Americans, it was not seen in our cohort. About 60% of the children were referred to the CRTCA by pediatricians or brought by their own parents who were worried about their health. The remaining 40% were sent by the principals of public schools in Campos, after personal contact to explain the aim of the study. The reason for sending children who were slim was to check whether their weight or height was normal. Overweight/obese children were sent for this reason or because dyslipidemia had been found on initial screening by a pediatrician. Children with thyroid, kidney or liver disorders, psychiatric diseases, non-essential hypertension or acute or chronic infections, who were using any medication that could interfere with the variables analyzed, or who were undergoing treatment for weight control were excluded. According to these criteria three children were excluded because they presented levels of CRP higher than 8 ng/dL, suggesting an acute infection. The study was performed according to Helsinki Declaration and approved by the Ethical Committee in Research of the University of São Paulo (203/2011). The individuals had their identity preserved and participated in the study only after the Terms of Consent had been signed by their parents or guardians.

All subjects were examined by the same observer, who evaluated their weight (measured to the nearest 0.1 kg on a Filizola adult-type scale with the subject wearing light clothes, without shoes and with their feet juxtaposed) and height (measured to the nearest 0.1 cm on a wall-mounted stadiometer of Halpender–Holtain type (Tonelli)) [7]. WC was measured in centimeters with an inelastic tape just above the superior border of the right iliac crest at the end of normal expiration, according to the NHANES III study [7]. These methods were chosen because the original NHANES III included a large sample of children from different ethnic groups (including African Americans) that closely resembled our sample. Systolic blood pressure (SBP) and diastolic blood pressure (DBP) were measured in millimeters of mercury in the right upper limb with a cuff appropriate to the length and circumference of the arm, after the child was rested and had been sitting down for at least five minutes. Blood pressure (BP) was checked twice during the appointment and the mean of the two measurements was used for analysis [8]. Values over 90th percentile (p90) were considered abnormal [9]. BP *z* scores were also calculated based on the reference values for sex, age and height [9]. BP was checked again at the second appointment about 45 days after the first evaluation, when the children returned with the results of the complementary tests requested. BMI *z* scores for all children were calculated from the 2007 WHO reference data [10], which classified them as normal-weight (*z* score > −1 and < 1) or overweight/obese (*z* score ≥ 1).

### Laboratory evaluation

At the first clinical evaluation, parents or guardians of all children were instructed to take their children to the CRTCA to get their blood drawn after 12-hour overnight fasting for the measurement of fasting glycemia, total cholesterol (TC), LDL, TG, HDL, insulin, CRP and leukocyte count. The assays were run in the Municipal Hospital of Guarus, in Campos. LDL was calculated using Friedewald’s formula [11]. Lipid profile values were established according to the 1st Brazilian Guidelines on the prevention of atherosclerosis in childhood and adolescence which consider normal levels to be TC < 150 mg/dl, LDL < 100 mg/dl, TG < 100 mg/dl and HDL ≥ 45 mg/dl [12]. The cut-off value for the Homeostatic Model of Assessment – Insulin Resistance (HOMA-IR) was ≥ 2.5 based on the study of Madeira [13]. Glycemia and lipid profiles were determined by the colorimetric enzymatic method using a kit for the Hitachi–Roche modular system. Insulin was measured by chemiluminescence using a Bayer kit, and CRP by immunonephelometry with a Dade Behring kit (Bayer). Leukocyte count was determined using an automated data processing system (Pentra 180 ABX).

#### Statistical analysis

BP values and metabolic parameters were compared among groups using Student’s *t*-test for continuous variables with a normal distribution and the Mann–Whitney test for those without a normal distribution. BMI *z* scores were calculated according to the 2007 WHO standard using Anthro Plus software. The sensitivity and specificity of BMI *z* score for the detecting changes in BP or HOMA-IR were calculated by receiver operating characteristic (ROC) curve analysis. The most sensitive WHtR cut-off value for the detection of cardio-metabolic changes was also determined using a ROC curve. Correlation analysis was conducted using Pearson or Spearman coefficients; the latter were used when the data were not normally distributed. Insulin and HOMA-IR values were not normally distributed on the Kolmogorov–Smirnof test and it was necessary to transform them into logarithms to evaluate their correlation with WHtR. Medcalc version 12.1.0.0, GraphPad Instat version 3.00 and Graph Pad Prism 5 version 5.04 were used for statistical analysis.

## Results

Of the 175 children 88 were classified as normal-weight and 87 as overweight/obese. The mean age with standard deviation of the two groups was 8.08 ± 1.24 years and 8.28 ± 1.15 years, respectively. The numbers of girls and boys were 40/48 and 37/50 for the normal-weight and the overweight/obese groups, respectively. In normal-weight group 41/88 were white and 47/88 of African American descent; in overweight/obese group 43/87 were white and 44/87 of African American descent. Of the 175 children 97 were African American descent (55%). The demographic and clinical data of children are shown in Table 1.

**Table 1:** Demographic and clinic data of normal-weight and overweight/obese 6-10-year-old children from Campos

**Group**	**Normal-weight**	**Overweight/obese**	**Statistical significance**
	**n = 88**	**n = 87**	
Girls	40 (45%)	37 (43%)	p = 0.8122
White	41 (47%)	43 (49%)	p = 0.7630
Age (median ± SD)	8.08 ± 1.24	8.28 ± 1.15	p = 0.2703
WHtR (median ± SD)	0.45 ± 0.004	0.58 ± 0.007	p < 0.0001
Height/age z score	−0.31 ± 1.08	0.84 ± 1.17	p < 0.0001
Waist (cm, median ± SD)	56.8 ± 5.22	76.5 ± 10.80	p < 0.0001
BMI z score (median ± SD)	−0,16 ± 0.64	2.90 ± 0.13	p < 0.0001
SBP z score (median ± SD)	−0.91 ± 1.12	0.94 ± 0.16	p < 0.0001
DBP z score (median ± SD)	0.32 ± 0.85	1.71 ± 1.15	p < 0.0001

There was a significant correlation between BP values recorded at the first and second appointments (*r* = 0.95 and *r* = 0.91 for SBP and DBP, respectively). Mean WHtR differed significantly between the normal-weight and overweight/obese groups (0.45 ± 0.004 and 0.58 ± 0.007 respectively, *p* < 0.0001). There was no difference between the mean WHtR in girls (*n* = 77) and that in boys (*n* = 86) (0.51 ± 0.009 and 0.52 ± 0.009 respectively; *p* = 0.53). There was a significant correlation between WHtR and BMI *z* score (*r* = 0.88, *p* < 0.0001). As shown in Table 2 WHtR was significantly correlated with all cardio-metabolic parameters except glycemia. Among the inflammatory markers WHtR was correlated with CRP (*r* = 0.51, *p* < 0.0001), but not with leukocyte count (*r* = 0.019, *p* = 0.805).

**Table 2:** Correlations between WHtR and cardio-metabolic parameters in normal-weight and overweight/obese 6-10-year-old children from Campos

**Metabolic variable**	***r *(CI 95%)**	***R***^**2**^	***p value***
HOMA-IR	0.83 (0.77 – 0.87)	0.68	< 0.0001
Insulin	0.79 (0.74 – 0.85)	0.64	< 0.0001
LDL	0.25 (0.11– 0.39)	0.06	0.0008
HDL	−0.28 (−0.41– 0.13)	0.08	0.0002
TG	0.26 (0.11– 0.39)	0.07	0.0006
Glycemia	0.14 (0.08 – 0.28)	0.02	0.063

The sensitivity of WHtR was similar to that of BMI *z* score for detecting at least one metabolic change (HOMA-IR, BP, LDL, HDL or TG): the respective areas under the curves (AUCs) were 0.739 and 0.717. As shown in Table 3 there were no differences between the AUCs for WHtR and BMI with respect to SBP, DBP, HOMA-IR, LDL and HDL. Neither WHtR nor BMI was able to detect hypertriglyceridemia. Tables 4 and 5 show the sensitivity and specificity of WHtR and the 2007 WHO BMI *z* score for screening the cardio metabolic alterations with their respective cut-off values. The most sensitive WHtR cut-off for HOMA-IR was >0.47, which was also capable of detecting any one of the cardio-metabolic disturbances (LDL, HDL, TG or BP) when they were accounted together. In the normal-weight group 29/88 (33%) had WHtR > 0.47, whereas in the overweight/obese group the proportion was 97% (84/87).

**Table 3:** **AUCs for WHtR and 2007 WHO BMI *z *score for screening metabolic risk factors in normal-weight and overweight/obese 6-10-year-old children from Campos**

**Metabolic risk**	**AUC**			
	**WHtR**	**p value**	**BMI *z *score**	**p value**
HOMA-IR	0.90 (0.83–0.95)*	0.0001	0.87 (0.81–0.92)*	0.0001
SBP	0.77 (0.70–0.83)*	0.0001	0.80 (0.73–0.87)*	0.0001
DBP	0.80 (0.73–0.85)*	0.0001	0.80 (0.73–0.86)*	0.0001
LDL	0.62 (0.54–0.69)*	0.0062	0.63 (0.55–0.70)*	0.0024
HDL	0.68 (0.61–0.75)*	0.0001	0.67 (0.59–0.74)*	0.0001
TG	0.58 (0.50–0.66)*	0.1002	0.53 (0.46–0.61)*	0.4997

* 95%CI: 95% confidence interval.

**Table 4:** Sensitivity and specificity of WHtR for screening metabolic risk factors in normal-weight and overweight/obese 6–10-year-old children from Campos

**Metabolicrisk**	**Sensitivity**	**Specificity**	**WHtR cut-off**
	**(95%CI)**	**(95%CI)**	
HOMA-IR	92.6 (75.7 – 99)	76.3 (65.2 – 85.3)	0.47
SBP	80.0 (64.4 –91)	68.9 (60.4 – 76.6)	0.51
DBP	76.6 (66 – 85.5)	74.5 (64.7 – 83)	0.49
LDL	65.8 (54 – 76.3)	55.0 (44.2 – 64.6)	0.48
HDL	60.0 (49 – 70.5)	70.0 (59.4 – 79.2)	0.50
TG	66.1 (53 – 77.7)	45.1 (35.8 – 54.8)	0.47

**Table 5:** **Sensitivity and specificity of the 2007 WHO BMI *z *score for diagnosing metabolic risk factors in normal-weight and overweight/obese 6-10-year-old children from Campos**

**Metabolic risk**	**Sensitivity**	**Specificity**	**BMI *z *score**
	**(95% *C *I)**	**(95%CI)**	
HOMA-IR	88 (68.8 – 97.5)	80.0 (72.7 – 86.1)	2.26
SBP	87.5 (70.2 – 94.3)	68.9 (60.4 – 76.6)	1.39
DBP	79 (68.5 – 87.6)	76.6 (66.9 – 84.5)	1.12
LDL	64.5 (52.7 – 75)	56.6 (46.2 – 66.5)	0.82
HDL	54.1 (43 – 65)	76.7 (67 – 84.9)	1.82
TG	50.0 (37 – 63)	59.3 (49.6 – 68.4)	1.39

## Discussion

Obesity in childhood is an important risk factor for the development of atherosclerotic heart disease [14] once an increased BMI from 10 years of age is considered the strongest predictor of premature death by acute myocardial infarction during adult life [15]. Insulin resistance (IR) seems to have an important role in the pathogenesis of atherosclerosis and metabolic syndrome which are related to overweight and mainly to abdominal adiposity [14,16].

BMI is the most traditional anthropometric index used for diagnosing overweight. As the prevalence of childhood obesity has grown up, the WHO reviewed the 2000 Centers for Disease Control (CDC) BMI curves to increase their sensitivity for the diagnosis of overweight [10], producing the 2007 WHO BMI standard, which is the most sensitive reference available at present. To our knowledge this is the first study comparing WHtR to this BMI reference for the assessment of cardio-metabolic risk factors, since all others have used the 2000 CDC standard [17,18]. We found WHtR to be capable of detecting an increase in LDL at a cut-off value of > 0.48 which would not have been possible using the WHO BMI only, as children with this WHtR are considered lean using the WHO BMI, because they are classified in the BMI *z* score < 0.8. Such findings are in agreement with those of other population-based studies that reinforce the importance of including WHtR measurements in routine care, with the aim of detecting the presence of cardio-metabolic risk factors even in normal-weight children [6,17,19,20].

We found a strong correlation between WHtR and the WHO BMI standard with respect to the assessment of weight (*r* = 0.88). This finding was close to that reported by Savva et al. (*r* = 0.91), who studied adolescents aged 10–14 years [4]. Regarding BP, in the study of Hara et al. [21] the correlation between WHtR and SBP (*r* = 0.27) and DBP (*r* = 0.25) was lower than that suggested by our results. Although WHtR was strongly correlated with HOMA-IR in the present study, this was not the case for glycemia, probably because IR is incipient in 6–10-year-old children.

The AUCs for WHtR and BMI in our study were similar to those observed by Freedman et al. [22] for screening elevated LDL (0.62 and 0.61 for WHtR and BMI respectively) and HDL (0.68 and 0.64 respectively). However, for screening SBP and DBP alterations the WHtR AUCs found by Freedman were lower than ours (0.68 and 0.54 for SBP and DBP, respectively). We believe that the differences between our results and those of Freedman [22] and Hara [20] regarding BP are a consequence of a higher proportion of African American descent in our sample from Rio de Janeiro, when compared to those from USA and Japan, as hypertension is more prevalent and severe in this ethnic group and in Latin American people [23]. The population-based samples studied by Freedman, for example, comprised 57% of whites [22] while our sample had 55% of African American descent.

Both anthropometric indexes were unable to diagnose hypertriglyceridemia either because IR is still incipient at this age or due to their ethnicity as people of African descent have lower serum TG levels [23].

In the present study the correlation with and sensitivity of WHtR were maximal for IR and hypertension. This leads us to believe that IR is one of the earliest metabolic disturbances to arise and contributes to hypertension, stimulating sodium and water renal retention, the sympathetic nervous system and vasoconstriction [14]. Through IR the lypolysis is intensified in adipose tissue, creating atherogenic dyslipidemia which together with the production of cytokines by visceral adipose tissue promote inflammatory reactions and hepatic CRP synthesis [24,25].

We would like to highlight that until we know this is the first study to demonstrate a relationship between WHtR and inflammatory markers. WHtR has previously been related only to CRP indicating that endothelial inflammation may already be evident in 6–10-year-old children. In contrast to the findings of a study of older children (8–16 years) [25], the absence of correlation between WHtR and leukocyte count in our study suggests that in 6–10-year-old children this inflammatory process is mediated by adipokines only.

The International Diabetes Federation (IDF) established ethnic and sex-specific cut-off points for WC indicative of metabolic risks in adults [26]. In children under 10 years however, there is no specific cut-off for WC or BMI that defines abdominal obesity and/or cardio-metabolic risk [27-29]. This is the reason why WHtR has become an attractive and practical option in recent years [30]. The 0.5 cut-off value brings adults and children, men and women closer together and allows the characterization of visceral fat even in normal-weight individuals [16,18], depending only on a tape measure and height measurement [5,31]. Some authors have questioned the use of this cut-off point as a universal indicator of cardio-metabolic risk [32], arguing that Asian populations tend to be shorter than Caucasians and as a consequence, the risk in the former would be increased at a lower WC [33]. Others, however, did not find any difference between the sexes, ethnic or age groups [16] because the division of WC by height aims to minimize such differences [34]. Despite this disagreement cardiovascular risk factors tend to group together more frequently above 0.5 constituting the metabolic syndrome [35], as we observed in the present study. Another advantage of WtHR is the individualization of the WC cut-off value with respect to height as an indicator of risk: using a cut-off value of less than one-half of the individual’s height, shorter adults could be shown to be at risk without those cut-off points proposed by the IDF. This value could also be used to screen for metabolic risk early, even in children under 10.

We would like to highlight some limitations of our study. The first was that we did not have access to data on other factors that could influence BP, such as family history of hypertension, salt intake and the eating habits of the population under study.

The second limitation was related to the use of the HOMA-IR for diagnosing IR. Use of the gold standard, the euglycemic hyperinsulinemic clamp, is not feasible in children because it is a complex procedure, difficult to perform and expensive. The HOMA-IR is an alternative which requires only fasting glucose and insulin measurements [35]. Some authors argue that there is no consensus regarding the ideal HOMA-IR cut-off value in children, and the commercially available kits also measure pro insulin [36]. However, the HOMA-IR is reliable and practical since it is standardized for a specific population as we chose, based on that of Madeira’s study, whose data came from a cohort of children of similar age to ours and originating from Rio de Janeiro [12]. Furthermore, results on the HOMA-IR are strongly correlated with those obtained using the euglycemic hyperinsulinemic clamp (*r* = 0.82) and are thus considered reliable for epidemiologic studies on a large scale [36]. Regarding the kits used to measure insulin, the cross-reaction with pro insulin is small (estimated in 8% for the chemiluminescence test).

Finally we would like to emphasize that our sample comprised children from low-income families and public schools in the north of Rio de Janeiro. Therefore, we suggest caution in the extrapolation of our results. As the aim is to call attention to the correlation between an easy anthropometric method (WHtR) for screening the presence of cardio-metabolic risk factors, we suggest that further population-based studies should be carried out for the exploration of the observed associations.

To prevent cardiovascular disease and DM2 increasing on a large scale the message “keep your waist to less than half your height” is simple and can be effective in public health education [37]. It also indicates that each one of us has an individual critical WC value that can be more effective in screening for metabolic risk than a single cut-off value pre-established for everyone, as has been proposed recently [37].

## Conclusions

WHtR is as sensitive as BMI in screening for cardio-metabolic and inflammatory risk. It is feasible, has a low cost and the ability to explain abdominal adiposity and is able to detect cardio-metabolic risk even in lean children. The message “keep your WC to less than half your height” can be useful for cardio-metabolic risk prevention programs, with the advantage of individualizing WC cut-point and metabolic risk.

## Competing interests

The authors declare that they have no competing interests.

## Authors’ contributions

VMK: the principal investigator, conceived, implemented and prepared the manuscript. CL: contributed to the study design, statistical analysis and interpretation of the results and helped to draft the manuscript. DD: contributed to the study design, interpretation of the results and coordination of the project. All authors read and approved the final manuscript.

## Pre-publication history

The pre-publication history for this paper can be accessed here:

http://www.biomedcentral.com/1471-2431/13/91/prepub
